# Tuberculosis-related knowledge, attitudes, and self-reported preventive practices among medical students at the University of Khartoum: A cross-sectional educational survey

**DOI:** 10.1371/journal.pone.0357003

**Published:** 2026-08-25

**Authors:** Mohammed Hamid, Mohamedelghali Elshaikh, Omer Yousif Abdelrahman Mohamed

**Affiliations:** Faculty of Medicine, University of Khartoum, Khartoum, Sudan; King Abdulaziz University Faculty of Medicine, SAUDI ARABIA

## Abstract

**Background:**

Tuberculosis (TB) remains the leading infectious cause of death from a single pathogen, disproportionately affecting low- and middle-income countries. Sudan’s TB burden has been compounded by prolonged armed conflict and deteriorating health infrastructure, yet no published study has assessed TB-related knowledge, attitudes, and self-reported practices (KAP) among Sudanese medical students. This study assessed TB-related KAP among medical students at the University of Khartoum and explored sociodemographic predictors of KAP outcomes.

**Methods:**

A cross-sectional survey was conducted at the Faculty of Medicine, University of Khartoum, Sudan (August–October 2024), using a proportionally allocated stratified convenience sample of 311 undergraduate medical students from the third to sixth academic years (response rate 91.5%). A validated, self-administered questionnaire assessed sociodemographic characteristics, TB knowledge (10 items), attitudes (7-item Likert scale), and self-reported preventive practices (5 items). Associations between KAP domains were examined using Pearson correlation, and multiple linear regression identified sociodemographic predictors of each domain, adjusting for sex, age group, and academic year (SPSS version 29).

**Results:**

The mean knowledge score was 6.43 ± 2.52 out of 10 (64.3%), indicating moderate overall knowledge. Correct responses were common for TB etiology (94.9%) and airborne transmission (91.3%) but less common for latent TB non-infectiousness (29.3%) and tuberculin skin test (TST) interpretation (49.2%). The mean attitude score was 27.90 ± 4.27 out of 35, and the mean self-reported practice score was 19.55 ± 3.67 out of 25; only 28.3% reported consistently wearing a face mask when interacting with suspected or confirmed TB patients. Knowledge correlated weakly with attitude scores (r = 0.179, p = 0.001) and was not significantly correlated with practice scores (r = 0.090, p = 0.113); attitude and practice scores were moderately correlated (r = 0.437, p < 0.001). Among the sociodemographic variables assessed, academic year was the strongest predictor of knowledge scores (adjusted R² = 0.474, p < 0.001), whereas the attitude and practice models explained minimal variance (adjusted R² = 0.029 and 0.018, respectively); female sex was the only significant predictor of self-reported practice (B = 0.95, 95% CI: 0.09–1.80, p = 0.031).

**Conclusions:**

In this single-institution study, medical students at the University of Khartoum demonstrated moderate knowledge of core tuberculosis concepts, with particular strength in disease causation and transmission but important gaps in latent tuberculosis infection and tuberculin skin test interpretation. Knowledge was weakly associated with attitudes and was not associated with self-reported preventive practices, suggesting that factual knowledge alone may not be sufficient to promote corresponding behavior. Given the convenience-sampling design and single-institution setting, these findings should be interpreted as an educational snapshot rather than generalized to all Sudanese medical students; they may nonetheless inform curriculum discussions on reinforcing tuberculosis-related knowledge, attitudes, and preventive practices.

## Introduction

Tuberculosis (TB), caused by Mycobacterium tuberculosis, primarily affects the lungs, though extrapulmonary disease involving the lymph nodes, pleura, and central nervous system is well recognized [1,2]. In 2024 alone, TB caused an estimated 1.23 million deaths, remaining the leading infectious cause of mortality from a single pathogen [3]. Despite the WHO End TB Strategy launched in 2014–2015, the global decline in TB incidence between 2015 and 2023 was only 8.3%, far short of the 50% interim target set for 2025 [4,5]. TB disproportionately affects socioeconomically disadvantaged populations, and in high-burden settings, limited awareness of TB symptoms and preventive measures contributes to delayed care-seeking and continued transmission [6–8].

Sudan faces a particularly acute TB burden in the context of prolonged political instability, recurrent armed conflict, and a deteriorating health infrastructure. The country is currently experiencing an escalating humanitarian crisis, with millions of people affected by severe physical and mental health challenges and large populations of internally displaced persons and refugees [9,10]. The armed conflict in Sudan between 2023 and 2024 was associated with a significant rise in TB incidence, from 54 to 63 cases per 100,000 population, alongside higher rates of recurrent TB, poor adherence to treatment regimens, and an expanding burden of multidrug-resistant TB (MDR-TB) [11]. This context profoundly shapes the environment in which future health professionals are trained and will subsequently practice.

Medical students represent a high-risk group for TB exposure: as they progress through clinical training they increasingly encounter undiagnosed and infectious patients in crowded ward and outpatient settings, often before receiving formal infection-control training, and their eventual clinical decisions will directly shape case detection and community transmission. In Sudan, the 2023–2024 conflict has disrupted formal teaching schedules, clinical rotations, and infection-control resources at several medical faculties, potentially altering the timing and consistency of TB-related instruction relative to the pre-conflict curriculum. Assessing KAP in this population is expected to support TB control not only by identifying curricular gaps before students enter independent practice, but also by clarifying whether attitudes and self-reported preventive behaviors keep pace with factual knowledge, since prior KAP research has repeatedly shown that these domains do not necessarily move together [12,13]. Most published TB KAP studies among health-professional students have been conducted in comparatively stable, well-resourced settings [14–16]; recent work has begun to extend KAP assessment to other high-burden, resource-limited settings such as Liberia [17], but the present study differs from all of these in assessing a cohort training through an active humanitarian crisis, where curricular disruption and constrained clinical resources may plausibly widen, rather than narrow, the knowledge–practice gap.

To the authors’ knowledge, no published study has assessed TB-related KAP among medical students in Sudan. Given Sudan’s high TB burden, the conflict-related disruption of medical education, and the general paucity of KAP evidence from active conflict settings, this study aimed to assess TB-related knowledge, attitudes, and self-reported preventive practices among medical students at the Faculty of Medicine, University of Khartoum, and to identify sociodemographic predictors of these outcomes.

## Materials and methods

### Study design

This descriptive cross-sectional study was conducted at the Faculty of Medicine, University of Khartoum, Sudan, from August to October 2024.

### Study area

The study was conducted at the Faculty of Medicine, University of Khartoum, Sudan, one of the oldest and most prominent medical institutions in the country. The faculty offers a six-year undergraduate medical program structured into preclinical and clinical phases. Clinical exposure, including training in infectious diseases such as tuberculosis, was introduced primarily during the later years of study, making this setting particularly relevant for evaluating students’ preparedness in TB-related knowledge and practice.

### Study population

The study targeted undergraduate medical students enrolled in the clinical and senior preclinical phases of the MBBS program at the Faculty of Medicine, University of Khartoum, Sudan. Students from batches 95–98 were included, corresponding to third- to sixth-year levels of training (batch 98: third year; batch 97: fourth year; batch 96: fifth year; and batch 95: sixth year). Students in the first and second academic years (batches 99 and 100) were excluded, as their training is limited primarily to basic sciences with minimal exposure to clinical medicine and infectious disease management, including tuberculosis (TB). Restricting the sample to students in the third to sixth years ensured the inclusion of participants with relevant clinical exposure and sufficient academic background to meaningfully respond to TB-related knowledge, attitudes, and practices (KAP) items.

### Sample size calculation

The required sample size was calculated using the finite population correction (FPC) formula for a known population:


$$\begin{array}{c} \text{n }=\;[\text{N }\times \;{\text{Z}}^{\text{2}}\;\times \text{ P}(\text{1}-\text{P})]\;/\;[{\text{d}}^{\text{2}}\;\times \;(\text{N}-\text{1})\;+\;{\text{Z}}^{\text{2}}\;\times \text{ P}(\text{1}-\text{P})] \end{array}$$

where n is the required sample size, N is the total population size, Z is the standard normal deviate corresponding to the desired confidence level, P is the estimated proportion of the attribute in the population, and d is the acceptable margin of error.

The following assumptions were applied: total population N = 1,311 (total eligible students across batches 95–98), Z = 1.96 (95% confidence level), P = 0.50 (assumed in the absence of prior local estimates to yield the maximum sample size), and d = 0.05 (5% margin of error).

The total population (N = 1,311) was distributed across batches as follows: Batch 95 comprised 326 students (24.9%), Batch 96 comprised 319 students (24.3%), Batch 97 comprised 346 students (26.4%), and Batch 98 comprised 320 students (24.3%).

On the basis of these parameters, the minimum required sample size was 297 participants. To account for potential nonresponses and incomplete submissions, the questionnaire was distributed electronically to approximately 340 students. A total of 311 participants completed the survey, yielding a response rate of 91.5%, which exceeded the minimum required sample size.

### Sampling method

A proportionally allocated stratified convenience sample was used. The study population was stratified by academic batch, and proportional allocation was applied on the basis of the size of each batch. Within each stratum, participants were recruited through convenience sampling via online dissemination using a Google Forms link distributed through WhatsApp groups, email lists, and class representatives over four weeks. Because recruitment within strata was convenience-based rather than random, the findings should not be assumed to be fully representative of all students at the faculty.

Participation was voluntary. Electronic informed consent was obtained prior to questionnaire completion. All responses were collected anonymously. The survey platform’s single-response-per-respondent setting was enabled to reduce duplicate entries, and the dataset was additionally screened for duplicate response patterns (e.g., identical timestamps and response combinations) prior to analysis; the reported number of students approached (≈340) represents an approximate rather than exact sampling frame, since dissemination occurred across overlapping WhatsApp groups, email lists, and class-representative channels and some double-counting of the denominator cannot be fully excluded.

### Data collection instrument

Data were collected using a structured, self-administered questionnaire adapted, with contextual modifications for the Sudanese setting, from previously published instruments assessing knowledge, attitudes, and practices (KAP) toward tuberculosis among healthcare students [18,19]. The questionnaire was developed in English. The final instrument comprised four sections. The first section captured sociodemographic characteristics, including age, sex, and academic year. The second section assessed knowledge of tuberculosis through 10 items covering etiology, modes of transmission, clinical manifestations, diagnostic methods, treatment, and prevention; responses were recorded as correct or incorrect/don’t know. The third section evaluated attitudes using seven statements measured on a five-point Likert scale ranging from strongly disagree to strongly agree, with the single negatively worded item reverse-coded prior to analysis. The fourth section assessed self-reported preventive practices using five items rated on a five-point frequency scale (never to always), with a focus on practices such as respiratory hygiene, the use of appropriate respiratory protection during patient care, personal protective measures, and environmental ventilation; the questionnaire did not separately capture whether respondents had previously had direct clinical contact with a TB patient (see Limitations). The questionnaire was administered electronically via a secure online platform to facilitate accessibility and enhance response rates. Consistent with this design, re-verification against the raw response file (S1 Data) confirmed no missing values across any of the 311 analyzed responses; no questionnaires were excluded for incompleteness. The complete questionnaire is provided as Supporting Information (S2 File).

### Questionnaire validity and reliability

The questionnaire underwent a multistep validation process. Content validity was assessed by three experts in community medicine and infectious diseases, each with more than five years of clinical or academic experience and prior involvement in questionnaire development, who independently evaluated each item for relevance, clarity, and comprehensiveness using a four-point rating scale (1 = not relevant, 2 = somewhat relevant, 3 = quite relevant, 4 = highly relevant). Item-level Content Validity Index (I-CVI) values ranged from 0.78 to 1.00, and the scale-level index (S-CVI/Ave), calculated as the mean of all I-CVI values, was 0.91, indicating excellent content validity. Because Cohen’s kappa is defined for pairs of raters, pairwise inter-rater agreement was calculated for each of the three reviewer pairs and averaged, yielding a mean κ of 0.83, reflecting strong agreement. Minor revisions were made to item wording based on the reviewers’ qualitative feedback prior to pilot testing. Face validity and feasibility were then evaluated through a pilot study among 20 medical students at the University of Khartoum who were not included in the final sample; pilot participants assessed the clarity, comprehension, and feasibility of the items, and minor wording adjustments were made without altering the overall structure or item count. Internal consistency reliability, assessed using Cronbach’s alpha, was 0.78 for the knowledge section, 0.74 for the attitude scale, and 0.72 for the practice scale, indicating acceptable reliability. The complete questionnaire is provided as Supporting Information (S2 File) so that item wording, response options, and scoring can be independently reviewed.

### Calculation of KAP scores

The knowledge section (10 items) was scored by assigning one point per correct response and zero points for incorrect or ‘do not know’ answers, yielding a composite knowledge score on a 0–10 scale. For the multiple-response symptom item (Question 2), a student was scored as correct only if they selected all six correct symptoms and no incorrect options; this stringent all-or-nothing scoring was adopted to avoid inflating the knowledge score through partial credit for incomplete or over-inclusive symptom recognition, consistent with the scoring approach used in the source instruments [14,15]. The attitude section (7 items, 5-point Likert scale) yielded a composite score ranging from 7 to 35, with higher scores indicating more favorable attitudes. Item 3 (“I cannot imagine myself working with TB patients in the future”) was the only reverse-worded item and was reverse-scored prior to computing the composite, so that higher scores consistently reflected more favorable attitudes across all items. Practice scores were computed by summing the five frequency-scale items (range 5–25), with higher scores reflecting more frequent engagement in self-reported preventive practices. All three domain scores were retained as continuous numerical variables throughout the analysis.

### Bias control and ethical considerations

Proportional allocation across batches reduced selection bias in batch representation. The use of an anonymous online questionnaire was intended to reduce social desirability bias, though it cannot eliminate it entirely; self-reported responses may not fully reflect actual clinical behavior. Recall bias was limited by the focus on current knowledge and behaviors. The study was reviewed and approved by the Institutional Review Board (IRB) of the Department of Community Medicine, Faculty of Medicine, University of Khartoum (ID: COMMED 2024-95-17). Electronic informed consent was obtained from all participants before questionnaire completion. All participants were informed of the study objectives, confidentiality measures, and their right to withdraw at any stage without consequences.

### Statistical analysis

Data were coded, cleaned, and analyzed using SPSS version 29. Descriptive statistics summarized participant characteristics and KAP outcomes. Continuous variables are presented as means and standard deviations (SD); categorical variables are reported as frequencies and percentages.

Pearson correlation analysis was conducted to evaluate associations between knowledge, attitude, and self-reported practice scores. Because knowledge scores were derived from a bounded, discrete 0–10 count of correct responses, Spearman’s rank-order correlation was additionally computed for all KAP domain pairs as a robustness check; results were consistent in direction and significance with the Pearson correlations reported here, and Pearson coefficients are presented throughout for comparability with prior KAP literature [16–18,20,29–31]. Correlation coefficients (r) were interpreted using standard thresholds (weak: < 0.3, moderate: 0.3–0.5, strong: > 0.5).

Multiple linear regression was performed to identify independent predictors of each KAP domain score, treating knowledge (0–10), attitude (7–35), and self-reported practice (5–25) as continuous numerical outcomes. Three covariates were included: sex (male = reference), age group (≤22 years = reference), and academic batch (Batch 98, 3rd year = reference), with Batches 97, 96, and 95 entered as dummy variables. Although age was recorded as a continuous variable, it was dichotomized for regression modeling to reflect a clinically interpretable split approximating the transition between earlier and later clinical years; continuous-age sensitivity models were not performed, and this simplification is noted as a limitation. Variance inflation factors (VIFs) were computed for all predictors to assess multicollinearity; all were below 4.0. For each model, residual normality was assessed by visual inspection of P-P plots, homoscedasticity by plots of standardized residuals against predicted values, and influential observations by Cook’s distance (threshold > 1); no substantial violations of model assumptions or highly influential cases were identified. Results are reported as unstandardized coefficients (B) with standard errors (SE), 95% confidence intervals (CI), t-statistics, and p-values. Statistical significance was set at p < 0.05 (two-tailed).

## Results

### Sociodemographic characteristics

A total of 311 medical students participated in this study, yielding a response rate of 91.5% among those approached. Among the participants, 107 (34.4%) were male and 204 (65.6%) were female. The majority of the participants were aged 22 years and less (166, 53.4%), whereas 145 (46.6%) were aged more than 22 years. Participants were distributed across academic batches as follows: Batch 98 (3rd year; n = 76, 24.4%), Batch 97 (4th year; n = 81, 26.1%), Batch 96 (5th year; n = 76, 24.4%), and Batch 95 (6th year; n = 78, 25.1%).

Previous awareness of tuberculosis was reported by 305 participants (98.1%). Formal education was the most frequently reported source of TB-related information (276, 88.7%), followed by the internet (133, 42.8%), healthcare providers (119, 38.3%), social media (110, 35.4%), television (47, 15.1%), and other sources (31, 10.0%). Participant characteristics are presented in Table 1.

**Table 1:** Sociodemographic characteristics and sources of tuberculosis-related information among participants (N = 311).

Variable	Category	Frequency (n)	Percentage (%)
**Sex**	Male	107	34.4%
	Female	204	65.6%
**Age (years)**	≤22 years	166	53.4%
	>22 years	145	46.6%
**Academic batch/Year of study**	Batch 98 (3rd year)	76	24.4%
	Batch 97 (4th year)	81	26.1%
	Batch 96 (5th year)	76	24.4%
	Batch 95 (6th year)	78	25.1%
**Previous awareness of TB**	Yes	305	98.1%
	No	6	1.9%
Sources of TB information*	Formal education	276	88.7%
	Internet	133	42.8%
	Healthcare providers	119	38.3%
	Social media	110	35.4%
	Television	47	15.1%
	Other sources	31	10.0%

*Multiple responses allowed; percentages do not sum to 100%.

### Knowledge regarding tuberculosis

The mean composite knowledge score was 6.43 ± 2.52 out of 10 (64.3%), equivalent to moderate rather than strong overall knowledge. The highest proportion of correct responses was observed for identifying bacteria as the causative agent (295, 94.9%; 95% CI: 92.5–97.3%) and airborne transmission (284, 91.3%; 95% CI: 88.2–94.4%). Correct responses were also observed for BCG vaccination at birth (214, 68.8%; 95% CI: 63.7–73.9%), first-line treatment (210, 67.5%; 95% CI: 62.3–72.7%), primary diagnostic method (188, 60.5%; 95% CI: 55.1–65.9%), and treatment duration (181, 58.2%; 95% CI: 52.7–63.7%). Lower correct response rates were observed for the anatomical location of pulmonary lesions (150, 48.2%; 95% CI: 42.6–53.8%), tuberculin skin test interpretation (153, 49.2%; 95% CI: 43.6–54.8%), and the recognition that patients with latent TB cannot transmit the disease (91, 29.3%; 95% CI: 24.2–34.4%). For the multiple-response symptom item, only 99 participants (31.8%; 95% CI: 26.6–37.0%) correctly selected all six symptoms with no incorrect options. Item-level responses, including 95% confidence intervals for the correct-response rate of each item, are presented in Table 2.

**Table 2:** Distribution of responses to individual knowledge items, with 95% confidence intervals for correct-response rates (N = 311).

Question / Response option	N	%	95% CI (correct item)
**1. What causes TB?**			
Bacteria (correct)	295	94.9%	92.5–97.3%
Virus	8	2.6%	
Fungus	1	0.3%	
Don’t know	7	2.3%	
2. Common symptoms of pulmonary TB (multiple responses allowed)*			
Fever (correct)	232	74.6%	
Rash	34	10.9%	
Cough lasting more than 2 weeks (correct)	270	86.8%	
Weight loss and loss of appetite (correct)	256	82.3%	
Blood in sputum (correct)	255	82.0%	
Chest pain (correct)	169	54.3%	
Night sweats (correct)	223	71.7%	
Abdominal pain	27	8.7%	
All 6 correct symptoms selected, no incorrect options	99	31.8%	26.6–37.0%
**3. How can a person get TB?**			
Through air droplets during coughing or sneezing (correct)†	284	91.3%	88.2–94.4%
Through handshakes	1	0.3%	
Orally through unclean food and water	8	2.6%	
Don’t know	18	5.8%	
**4. In which region of the lung do pulmonary TB lesions typically occur?**			
Lower lobes	57	18.3%	42.6–53.8%
Middle lobes	16	5.1%	
Upper lobes (correct)	150	48.2%	
Don’t know	88	28.3%	
**5. Patients with latent tuberculosis can spread the disease:**			
True	133	42.8%	24.2–34.4%
False (correct)	91	29.3%	
Don’t know	87	28.0%	
**6. What is the most appropriate diagnostic method for pulmonary TB?**			
Blood test	18	5.8%	55.1–65.9%
Sputum test (correct)	188	60.5%	
Chest X-ray	57	18.3%	
Urine/stool test	2	0.6%	
Don’t know	46	14.8%	
**7. What does a positive tuberculin skin test for TB indicate?**			
Indicates a latent TB infection only	29	9.3%	43.6–54.8%
Indicates active TB disease only	14	4.5%	
Indicates either a latent TB infection or active TB disease (correct)	153	49.2%	
Don’t know	115	37.0%	
**8. BCG vaccine is administered to newborns as part of their vaccination schedule:**			
True (correct)	214	68.8%	63.7–73.9%
False	15	4.8%	
Don’t know	82	26.4%	
**9. What is the mainstay of treatment for most patients with pulmonary tuberculosis?**			
Ciprofloxacin	8	2.6%	62.3–72.7%
Isoniazid, rifampicin, pyrazinamide, ethambutol (correct)	210	67.5%	
Ceftriaxone, cefixime	6	1.9%	
Don’t know	87	28.0%	
**10. How long does it take to treat TB?**			
1–2 weeks	5	1.6%	52.7–63.7%
1–2 months	13	4.2%	
6–8 months (correct)	181	58.2%	
1–2 years	26	8.4%	
Don’t know	86	27.7%	

*For question 2, a full-score response required selection of all six correct symptoms (fever, cough >2 weeks, weight loss, blood in sputum, chest pain, night sweats) and no incorrect options. Percentages for individual symptom options reflect all selections and do not sum to 100%. †Item wording used “air droplets”, consistent with phrasing at the time of instrument development; current WHO guidance describes TB transmission as airborne (aerosol) spread (see Limitations). 95% CIs (normal approximation) are reported for the correct-response rate of each item and for the overall symptom-recognition item.

### Attitudes toward tuberculosis

The mean composite attitude score was 27.90 ± 4.27 out of 35, with a median score of 29. The highest mean item scores were recorded for the importance of educating people about tuberculosis (4.35 ± 0.93), the importance of examining the contacts of TB patients (4.29 ± 0.92), and the belief that early detection and treatment improve outcomes (4.15 ± 0.86). The lowest mean score was recorded for the item “I cannot imagine myself working with TB patients in the future” (3.00 ± 1.13 before reverse coding). The item-level attitude responses are presented in Table 3.

**Table 3:** Distribution of responses to individual attitude items (N = 311).

Attitude question	Strongly Agree (%)	Agree (%)	Neutral (%)	Disagree (%)	Strongly Disagree (%)
1. I feel uncomfortable when anybody nearby sneezes or coughs without covering their mouth and nose	146 (46.9%)	107 (34.4%)	39 (12.5%)	8 (2.6%)	11 (3.5%)
2. A person should be examined for TB if there is a TB patient among their family or friends.	158 (50.8%)	110 (35.4%)	27 (8.7%)	8 (2.6%)	8 (2.6%)
3. I can’t imagine myself working with tuberculosis (TB) in the future.†	32 (10.3%)	72 (23.2%)	100 (32.2%)	77 (24.8%)	30 (9.6%)
4. I think TB can be cured if it is detected and treated early.	119 (38.3%)	139 (44.7%)	39 (12.5%)	9 (2.9%)	5 (1.6%)
5. Quarantine of TB patients may reduce the risk of the spread of TB.	103 (33.1%)	125 (40.2%)	60 (19.3%)	14 (4.5%)	9 (2.9%)
6. I believe that educating people about TB prevention methods is crucial for reducing the risk of TB and improving overall public health.	172 (55.3%)	103 (33.1%)	20 (6.4%)	6 (1.9%)	10 (3.2%)
7. TB-related stigma affects individuals’ willingness to seek medical care and treatment.	109 (35.0%)	118 (37.9%)	56 (18.0%)	17 (5.5%)	11 (3.5%)

†Reverse-scored prior to computing the composite attitude score (see Methods).

Because this item conflates several distinct constructs, including fear of infection, career preference, and perceived working conditions, the 33.5% of participants agreeing or strongly agreeing with it should not be interpreted as a single, validated measure of TB-related stigma or occupational reluctance; it is reported descriptively rather than as evidence of a unidimensional construct.

### Self-reported preventive practices

The mean composite self-reported practice score was 19.55 ± 3.67 out of 25 (median = 20). Covering the mouth while coughing or sneezing was reported as ‘always’ by 193 participants (62.1%) and ‘often’ by 81 (26.0%). Wearing a face mask or appropriate respiratory protection when caring for patients suspected or confirmed to have tuberculosis was reported as ‘always’ by 88 participants (28.3%) and ‘often’ by 90 participants (28.9%). Hand hygiene and use of personal protective equipment before contact with TB patients were reported as ‘always’ by 177 participants (56.9%) and ‘often’ by 76 (24.4%). Opening windows for ventilation was reported as ‘always’ by 143 participants (46.0%). Reading TB awareness materials was reported as ‘always’ by 65 participants (20.9%). Item-level responses are presented in Table 4.

**Table 4:** Distribution of responses to individual practice items (N = 311).

Practice question	Always (%)	Often (%)	Sometimes (%)	Rarely (%)	Never (%)
1. I cover my mouth when I cough or sneeze.	193 (62.1%)	81 (26.0%)	27 (8.7%)	8 (2.6%)	2 (0.6%)
2. I wear a face mask or other appropriate respiratory protection when caring for or interacting with patients suspected or confirmed to have TB.	88 (28.3%)	90 (28.9%)	70 (22.5%)	45 (14.5%)	18 (5.8%)
3. I perform hand hygiene and wear personal protective equipment before contact with pulmonary TB patients.	177 (56.9%)	76 (24.4%)	31 (10.0%)	14 (4.5%)	13 (4.2%)
4. I open windows when possible in TB patients’ rooms to increase natural ventilation.	143 (46.0%)	78 (25.1%)	60 (19.3%)	12 (3.9%)	18 (5.8%)
5. I read materials designed to raise awareness about TB and respiratory infections.	65 (20.9%)	69 (22.2%)	81 (26.0%)	63 (20.3%)	33 (10.6%)

Because the questionnaire did not separately assess whether participants had previously cared for a patient with confirmed or suspected TB, a response of ‘never’ on the practice items, particularly mask use, may in some respondents reflect the absence of relevant clinical exposure rather than a genuine preventive-practice deficit; this potential confound is addressed further in the Discussion and Limitations.

### Correlations between KAP domains

Pearson correlation analysis revealed a statistically significant positive correlation between knowledge and attitude scores (r = 0.179, p = 0.001), indicating that higher levels of tuberculosis (TB) knowledge were associated with more favourable attitudes. A moderate and highly significant positive correlation was observed between attitude and practice scores (r = 0.437, p < 0.001), suggesting that more positive attitudes toward TB were associated with better preventive practices. In contrast, the correlation between knowledge and practice scores was weak and not statistically significant (r = 0.090, p = 0.113). Spearman correlations for all three domain pairs were consistent in direction and significance with these Pearson results. The Pearson correlation analysis is presented in Table 5.

**Table 5:** Pearson correlation between knowledge, attitude, and practice scores (N = 311).

Variable pair	Pearson correlation (r)	p-value	Interpretation
Knowledge vs Attitude	0.179	0.001	Significant weak positive correlation
Attitude vs Practice	0.437	<0.001	Significant moderate positive correlation
Knowledge vs Practice	0.090	0.113	Nonsignificant weak correlation

### Multiple linear regression analysis of sociodemographic predictors of KAP outcomes

Multiple linear regression was performed to identify independent predictors of knowledge, attitude, and self-reported practice scores, adjusting simultaneously for sex, age group, and academic batch (Table 6). Batch 98 served as the reference category; all VIFs were below 4.0.

**Table 6:** Multiple linear regression: predictors of knowledge, attitude, and practice scores (N = 311).

Variable	B	SE	β	95% CI	t	p-value
**Outcome: Knowledge Score (0–10)** **R² = 0.483, Adjusted R² = 0.474; F(5,305) = 56.95, p < 0.001**						
Sex: Female (ref. Male)	−0.045	0.219	−0.008	−0.477, 0.386	−0.207	0.836
Age: > 22y (ref. ≤ 22y)	−0.484	0.319	−0.096	−1.111, 0.143	−1.520	0.130
4th Year, Batch 97 (ref. Batch 98)	2.258	0.301	0.394	1.666, 2.849	7.512	<0.001
5th Year, Batch 96	3.858	0.360	0.659	3.150, 4.566	10.723	<0.001
6th Year, Batch 95	5.136	0.424	0.885	4.301, 5.972	12.101	<0.001
**Outcome: Attitude Score (7–35)** **R² = 0.045, Adjusted R² = 0.029; F(5,305) = 2.87, p = 0.015**						
Sex: Female	0.280	0.505	0.031	−0.714, 1.274	0.554	0.580
Age: > 22y	−0.790	0.735	−0.092	−2.236, 0.656	−1.075	0.283
4th Year, Batch 97	−0.846	0.693	−0.087	−2.209, 0.518	−1.221	0.223
5th Year, Batch 96	0.669	0.829	0.067	−0.963, 2.301	0.806	0.421
6th Year, Batch 95	2.160	0.979	0.220	0.234, 4.086	2.207	0.028
**Outcome: Practice Score (5–25)** **R² = 0.034, Adjusted R² = 0.018; F(5,305) = 2.13, p = 0.022**						
Sex: Female	0.945	0.436	0.123	0.086, 1.803	2.166	0.031
Age: > 22y	−0.512	0.634	−0.070	−1.760, 0.737	−0.806	0.421
4th Year, Batch 97	−0.497	0.598	−0.060	−1.675, 0.680	−0.831	0.406
5th Year, Batch 96	0.319	0.716	0.037	−1.090, 1.728	0.446	0.656
6th Year, Batch 95	1.209	0.845	0.143	−0.454, 2.872	1.431	0.154

B = unstandardized regression coefficient; SE = standard error; β = standardized regression coefficient (computed as B × SD(predictor)/SD(outcome), using reported group proportions and outcome SDs); CI = confidence interval. Reference categories: male sex, age ≤ 22 years, Batch 98 (3rd year). All VIF values < 4.0.

The knowledge model was highly significant (R² = 0.483, adjusted R² = 0.474; F(5,305) = 56.95, p < 0.001). Academic batch was the strongest predictor: compared with Batch 98 (3rd year), students in Batch 97, 96, and 95 scored progressively higher, with mean differences of 2.26 points (95% CI: 1.67–2.85, p < 0.001), 3.86 points (95% CI: 3.15–4.57, p < 0.001), and 5.14 points (95% CI: 4.30–5.97, p < 0.001), respectively, on the 0–10 scale. Neither sex (B = −0.05, p = 0.836) nor age group (B = −0.48, p = 0.130) was a significant independent predictor of knowledge.

The attitude model was statistically significant overall (R² = 0.045, adjusted R² = 0.029; F(5,305) = 2.87, p = 0.015), though its explanatory power was modest. Only Batch 95 (6th year) had a significantly higher attitude score than Batch 98 (B = 2.16, 95% CI: 0.23–4.09, p = 0.028). Sex and age group were not significant predictors.

The self-reported practice model was statistically significant overall (R² = 0.034, adjusted R² = 0.018; F(5,305) = 2.13, p = 0.022), but explained a small proportion of total variance. Female sex was the only statistically significant predictor (B = 0.95, 95% CI: 0.09–1.80, p = 0.031), indicating that female students scored, on average, 0.95 points higher on the 5–25 self-reported practice scale than male students, independent of age and batch. No individual batch differed significantly from Batch 98 in self-reported practice scores (all p > 0.15), and age group was not significant.

## Discussion

The findings of this study offer a cross-sectional educational snapshot of TB-related knowledge, attitudes, and self-reported preventive practices among medical students at the University of Khartoum. Taken together, the results suggest that students have acquired foundational biomedical knowledge of TB through the early curriculum, but that this knowledge has not consistently translated into self-reported preventive practices, a pattern well-documented in the broader KAP literature [20].

The mean knowledge score was 6.43 ± 2.52 out of 10 (64.3%), representing moderate knowledge with important gaps rather than uniformly strong understanding. High correct response rates for the causative agent (94.9%) and airborne transmission (91.3%) are encouraging but expected; these are foundational concepts introduced early in the preclinical curriculum and are consistently well-retained in comparable studies across low- and middle-income countries [20–22]. The more informative findings concern items where correct responses were less frequent. Only 29.3% of participants correctly identified that patients with latent TB cannot transmit the disease, and fewer than half (49.2%) correctly interpreted the tuberculin skin test (TST). Both represent potentially consequential knowledge gaps in a clinical training context: misunderstanding latent infectiousness may affect how a student counsels household contacts of an index case, and difficulties with TST interpretation have been documented in comparable medical student populations in Pakistan and Saudi Arabia [14,15]. It should be noted, however, that this study did not assess actual clinical performance; these findings reflect self-reported questionnaire responses and should be interpreted as educational indicators rather than direct measures of clinical competence. The questionnaire’s transmission item used the term “air droplets,” consistent with commonly used phrasing at the time of instrument development; current WHO guidance more precisely describes TB transmission as airborne (aerosol) spread, and this terminology should be updated in future revisions of the instrument.

Symptom recognition findings also merit consideration. Only 31.8% of participants correctly selected all six classical TB symptoms with no incorrect options on the multiple-response item. Individually, commonly taught features such as prolonged cough (86.8%) and weight loss (82.3%) were well recognized, whereas chest pain was identified by 54.3% of participants. Incomplete symptom recognition has been documented in studies among medical students in Ethiopia and nursing trainees in India, and has been attributed to teaching approaches that emphasize the classical triad without sufficient coverage of the full clinical picture [23,24]. These patterns may suggest areas for targeted reinforcement within the curriculum, though the study design does not permit conclusions about the curriculum itself.

The mean attitude score was 27.90 ± 4.27 out of 35. Strong endorsement of the importance of contact tracing, public education, and early treatment reflects broad engagement with TB control principles, consistent with findings from student populations in Nigeria and Pakistan [25,26]. The item assessing occupational orientation toward TB, ‘I cannot imagine myself working with TB patients in the future’, yielded the lowest mean item score (3.00 ± 1.13 before reverse coding), with 33.5% of participants disagreeing or strongly disagreeing with this statement, indicating some degree of willingness, while 32.2% were neutral and 33.5% agreed or strongly agreed. Occupational reluctance among healthcare trainees toward TB care has been reported in multiple settings and has been associated with fear of infection and stigma-related discomfort; the literature suggests these attitudes are not automatically resolved by increased factual knowledge [27,28]. However, this single item reflects a complex and multidimensional construct, including concerns about working conditions, specialty preferences, and personal safety, and should not be over-interpreted as a definitive measure of occupational readiness.

More than 72% of participants recognized that TB stigma discourages care-seeking, a level of awareness that has educational value. The relationship between stigma awareness and actual clinical practice, however, is not straightforward; the ability to recognize that stigma exists does not necessarily translate into the communication and empathy skills needed to address it in a clinical encounter. Evidence from the stigma literature suggests that translating awareness into nonjudgmental clinical behavior requires deliberate, skill-based educational approaches [29].

The mean self-reported practice score was 19.55 ± 3.67 out of 25. Behaviors directly applicable to clinical settings, such as covering the mouth when coughing or sneezing (62.1% ‘always’) and reporting the use of personal protective equipment before TB patient contact (56.9% ‘always’), were relatively frequently self-reported. Wearing a face mask or appropriate respiratory protection when caring for patients with suspected or confirmed tuberculosis was reported as ‘always’ by 28.3% of participants, with 28.9% reporting ‘often’. This item directly captures infection-control behavior in a clinical training context, where consistent use of respiratory protection is considered standard precautionary practice. Lower frequencies of consistent masking during TB patient contact may reflect gaps in infection-control culture, limited availability of appropriate respiratory protection in training settings, insufficient reinforcement of occupational safety practices within the clinical curriculum, or simply limited direct clinical contact with TB patients among some respondents, though the cross-sectional, self-report design does not permit causal attribution or distinguish between these possibilities. Self-reported engagement with TB awareness materials was also modest, with 20.9% reporting they ‘always’ read such materials. As all practice items were self-reported, the scores may overestimate actual behavior due to social desirability bias, and do not represent observed clinical practice.

The correlation data offer structural context for these patterns. The association between knowledge and attitude scores was significant but weak (r = 0.179, p = 0.001), suggesting that higher factual knowledge is associated with marginally more favorable attitudes, though the relationship is modest. Knowledge and self-reported practice scores were not significantly correlated (r = 0.090, p = 0.113). This knowledge–practice gap has been documented across TB KAP studies in multiple student populations, including medical students in Hunan, China, general university students in Hainan, and students in Saudi Arabia [16,30–32], and is consistent with the broader observation that informational learning alone does not reliably shift protective behavior. The stronger association between attitude and self-reported practice scores (r = 0.437, p < 0.001) aligns with the Health Belief Model’s emphasis on perceived threat and attitudinal orientation as more proximal drivers of health-related behavior [33]. Taken together, these associations, based on a limited set of sociodemographic predictors in a single-institution cross-sectional design, should be interpreted as exploratory and not as evidence of causal relationships.

Academic year was the strongest predictor of knowledge scores among the limited set of sociodemographic variables assessed, explaining a substantial proportion of knowledge variance (adjusted R² = 0.474); by contrast, the attitude and practice models explained minimal variance (adjusted R² = 0.029 and 0.018, respectively), suggesting that factors not captured in this study are more important determinants of these outcomes. Compared with third-year students (Batch 98), students in higher academic years achieved progressively higher knowledge scores, with sixth-year students scoring more than five points higher on the 10-point scale after adjustment for sex and age (B = 5.14, 95% CI: 4.30–5.97, p < 0.001). This pattern likely reflects cumulative curricular and clinical exposure during medical training, consistent with findings from a similar study [30], and was reflected in the relatively high explanatory power of the knowledge model. Because these are cross-sectional comparisons across different academic cohorts rather than a longitudinal assessment of the same students over time, these differences should be interpreted as cohort-level associations and not as direct evidence of within-student educational improvement over the course of training.

For attitude scores, only sixth-year students had significantly more favorable scores than third-year students (B = 2.16, p = 0.028), while the overall model explained a modest proportion of variance, suggesting that factors beyond the sociodemographic variables assessed in this study contribute to attitudinal variation [34]. For self-reported practice, female sex was the only significant predictor (B = 0.95, 95% CI: 0.09–1.80, p = 0.031), consistent with reported sex differences in preventive health behavior in the literature [34,35]; however, the practice model explained only 1.8% of total variance, indicating that the predictors included were essentially uninformative for predicting practice scores and that the analysis was underpowered for the stated objective of identifying key sociodemographic predictors of practice. The regression models did not include important potential predictors such as prior TB patient contact, clinical rotation type, infection-control training history, or personal or family TB history, which limits the explanatory scope of these findings. These models should therefore be considered exploratory rather than confirmatory.

The prominence of the internet (42.8%) and social media (35.4%) as supplementary TB information sources is notable in an educational context. In a setting where formal teaching has been subject to disruption by conflict and where self-directed digital learning has increased, the accuracy of online TB content accessed by students may have curricular relevance. Integrating critical appraisal of digital health information into the medical curriculum may help students navigate unvetted sources, though this study did not directly measure the quality or content of students’ digital information use.

Taken together, these findings suggest that students at the Faculty of Medicine, University of Khartoum have acquired a foundational understanding of TB, particularly regarding etiology and transmission, but demonstrate notable gaps in latent TB concepts, TST interpretation, and the consistency of self-reported preventive practices. The absence of a significant association between knowledge and self-reported practice, and the limited association between academic year and practice scores, suggest that informational curricula may be insufficient on their own to promote consistent self-reported preventive practices. These observations may inform curriculum discussions regarding targeted reinforcement of specific knowledge gaps and the potential value of practice-oriented and attitudinally engaged educational approaches. Given the cross-sectional, self-report, and single-institution design of this study, however, causal conclusions cannot be drawn, and the generalizability of findings to other Sudanese medical schools is limited.

### Strengths and limitations

This study provides an educational assessment of TB-related KAP among medical students at the University of Khartoum, a setting for which no comparable published study currently exists. The high response rate of 91.5% and the use of proportional allocation across academic batches strengthened the internal distribution of the sample. The data collection instrument underwent multistep validation including expert content review, pilot testing, and internal consistency assessment (Cronbach’s alpha: 0.78 for knowledge, 0.74 for attitudes, 0.72 for practice).

Several important limitations must be acknowledged. First, the cross-sectional design precludes causal inference. Second, the study was conducted at a single institution; the findings reflect one medical faculty and should be interpreted as a single-institution educational snapshot rather than generalized to other Sudanese medical schools, which may differ in curriculum, resources, clinical exposure, and degree of conflict-related disruption. Third, within each academic batch, participants were recruited through convenience sampling via online dissemination, which limits representativeness; students with greater interest in TB or infectious diseases, or those with better internet access, may have been more likely to respond, and findings may not be representative of the full population of University of Khartoum medical students. Proportional allocation improved batch distribution but does not resolve this selection limitation. Fourth, all KAP data are self-reported; social desirability bias may lead to overestimation of favorable practices, and self-reported practice scores do not represent observed clinical behavior. Fifth, the regression models included only sex, age group, and academic batch; important predictors such as prior TB patient contact, infection-control training history, clinical rotation type, personal or family TB history, and digital information use were not captured, limiting the explanatory scope of the models. Sixth, the questionnaire’s transmission item used the term “air droplets” rather than the more precise current WHO terminology of airborne (aerosol) transmission; future revisions of the instrument should adopt updated terminology. Seventh, the practice scale did not separately capture prior direct clinical contact with TB patients, so low-frequency responses on practice items, particularly mask use, may partly reflect limited clinical exposure rather than a genuine preventive-practice deficit. Eighth, the attitude scale combined items addressing distinct constructs, including stigma, occupational willingness, and support for quarantine and public education, and a formal factor analysis to justify combining these items into a single composite score was not performed; the composite attitude score should be interpreted as a general orientation rather than a validated unidimensional construct. Ninth, age was collected as a binary category (≤22 vs > 22 years) at questionnaire design rather than as an exact value, so it could not be modeled continuously, which may obscure finer-grained age-related associations; future iterations of the instrument should collect exact age.

### Recommendations

The findings of this study may help inform curriculum discussions within the Faculty of Medicine, University of Khartoum. Particular attention could be directed toward areas where lower levels of knowledge were observed, including latent tuberculosis infection and tuberculin skin test interpretation, as well as aspects of self-reported preventive practices that showed lower levels of engagement. Given the absence of a significant association between knowledge and self-reported preventive practices, future educational initiatives may benefit from incorporating approaches that extend beyond factual instruction alone. Educational strategies such as case-based learning, simulation exercises, and supervised clinical training could be explored as potential methods for strengthening the application of tuberculosis-related knowledge in clinical and preventive contexts. However, the effectiveness of such approaches was not evaluated in the present study and warrants further investigation. The findings also suggest potential value in reinforcing topics related to infection prevention, occupational safety, and tuberculosis-related stigma within undergraduate medical education. Future multi-institutional studies should incorporate measures of prior clinical exposure to TB patients, use validated, factor-analyzed attitude instruments, and, where feasible, include observational or performance-based measures of practice rather than relying solely on self-report. Future research should evaluate whether targeted educational interventions are associated with improvements in knowledge retention, attitudes, and observed preventive practices. Given the increasing use of online and social media platforms as sources of health information, further research may also examine how students access, evaluate, and utilize tuberculosis-related information from digital sources and whether these sources influence educational outcomes.

## Conclusion

This study provides a single-institution educational snapshot of tuberculosis-related knowledge, attitudes, and self-reported preventive practices among medical students at the University of Khartoum. Participants demonstrated moderate overall knowledge of core tuberculosis concepts (mean score 64.3%), including disease causation and transmission, but showed lower levels of knowledge regarding latent tuberculosis infection and tuberculin skin test interpretation. Attitudes toward tuberculosis varied across the assessed domains, and some self-reported preventive practices were reported less consistently. A significant but weak association was observed between knowledge and attitudes, whereas knowledge was not significantly associated with self-reported preventive practices. These findings suggest that increasing factual knowledge alone may not necessarily translate into corresponding practices. Given the single-institution, convenience-sampling design, these results should not be generalized beyond this cohort. They may nonetheless help inform curriculum discussions regarding targeted reinforcement of latent tuberculosis concepts and practical infection-prevention training. Future multi-institutional studies incorporating validated attitude instruments and observational or performance-based measures of practice would provide a more comprehensive understanding of tuberculosis preparedness among medical students in Sudan.

## Supporting information

S1 Data Data of the research participants. (XLSX)

S2 File Questionnaire of the KAP toward TB research. (DOCX)

S3 File STROBE checklist for cross-sectional studies. (DOCX)

S4 File Completed human participants research checklist. (DOCX)

## References

[1] Natarajan A, Beena PM, Devnikar AV, Mali S. A systemic review on tuberculosis. Indian J Tuberc. 2020;67(3):295–311. doi: 10.1016/j.ijtb.2020.02.005 32825856

[2] Pattamapaspong N, Kanthawang T, Peh WCG, Hammami N, Bouaziz MC, Ladeb MF. Imaging of thoracic tuberculosis: pulmonary and extrapulmonary. BJR Open. 2024;6(1):tzae031. doi: 10.1093/bjro/tzae031 39363908 PMC11449295

[3] World Health Organization. Tuberculosis. https://www.who.int/news-room/fact-sheets/detail/tuberculosis 2025. Accessed 2025.

[4] World Health Organization. The End TB Strategy. https://www.who.int/publications/i/item/WHO-HTM-TB-2015.19 2015. Accessed 2025.

[5] World Health Organization. Global Tuberculosis Report 2024. Geneva: WHO. 2024. https://www.who.int/teams/global-programme-on-tuberculosis-and-lung-health/tb-reports/global-tuberculosis-report-2024

[6] Költringer FA, Annerstedt K, Boccia D, Carter DJ, Rudgard WE. The social determinants of national tuberculosis incidence rates in 116 countries: a longitudinal ecological study between 2005–2015. BMC Public Health. 2023;23(1):324. doi: 10.1186/s12889-023-15213-6 36793018 PMC9930041

[7] Carter DJ, Glaziou P, Lönnroth K, Siroka A, Floyd K, Weil D, et al. The impact of social protection and poverty elimination on global tuberculosis incidence: a statistical modelling analysis of Sustainable Development Goal 1. Lancet Glob Health. 2018;6(5):e514–22. doi: 10.1016/S2214-109X(18)30195-5 29580761 PMC5968370

[8] Saunders MJ, Evans CA. Fighting poverty to prevent tuberculosis. Lancet Infect Dis. 2016;16(4):395–6. doi: 10.1016/S1473-3099(15)00434-X 26725447

[9] Sudan Health Cluster. Sudan Health Cluster Annual Report 2021. 2022. https://reliefweb.int/report/sudan/sudan-health-cluster-annual-report-2021

[10] Khogali A, Homeida A. Impact of the 2023 armed conflict on Sudan’s healthcare system. Public Health Chall. 2023;2(4):e134. doi: 10.1002/puh2.134 40496780 PMC12039653

[11] Mohammed AK, Humida EH, Ali AM, Ahmed HG. Burden of Tuberculosis in Western Sudan During the Sudan Armed Conflict. Cureus. 2025;17(1):e76861. doi: 10.7759/cureus.76861 39906451 PMC11793838

[12] Craciun OM, Del Rosario Torres M, Benito Llanes A, Romay-Barja M. Tuberculosis knowledge, attitudes, and practice in middle- and low-income countries: a systematic review. Journal of Tropical Medicine. 2023;2023:1014666. doi: 10.1155/2023/1014666 37398546 PMC10314818

[13] Abu-Humaidan AHA, Tarazi A, Hamadneh Y, Al-Leimon A, Al-Leimon O, Aljahalin M, et al. Knowledge, attitudes, and practices toward tuberculosis among Jordanian university students. Front Public Health. 2022;10:1055037. doi: 10.3389/fpubh.2022.1055037 36478722 PMC9719926

[14] Ahmad S, Khawaja UA, Haider SM, Mowlabaccus WB, Mohan A, Ansari A, et al. Assessing the knowledge, attitude, and practice measures against tuberculosis in patients in ambulatory department facilities in Pakistan: a cross-sectional analysis. Monaldi Arch Chest Dis. 2023;94(1). doi: 10.4081/monaldi.2023.2500 37052048

[15] Kandasamy G, Almaghaslah D, Almanasef M. Knowledge, attitude and practice towards tuberculosis among healthcare and non-healthcare students at a public university in Saudi Arabia. Frontiers in Public Health. 2024;12:1348975. doi: 10.3389/fpubh.2024.1348975 38379677 PMC10877943

[16] Mohammed EA, Alotaibi HA, Alnemari JF, Althobiti MS, Alotaibi SS, Ewis AA, et al. Assessment of Knowledge, Attitude, and Practice towards Tuberculosis among Taif University Students. Healthcare. 2023;11(20):2807. doi: 10.3390/healthcare11202807 37893881 PMC10606274

[17] Terry AF, Etikan İ. Evaluating the Knowledge, Attitude, and Practice of Tuberculosis Among Health Sciences Students. Healthcare (Basel). 2025;13(13):1534. doi: 10.3390/healthcare13131534 40648559 PMC12248818

[18] Berg-Johnsen A, Hådem SO, Tamrakar D, Harstad I. A questionnaire of knowledge, attitude and practices on tuberculosis among medical interns in Nepal. Journal of Clinical Tuberculosis and Other Mycobacterial Diseases. 2020;20:100173. doi: 10.1016/j.jctube.2020.100173 32685706 PMC7358262

[19] Behnaz F, Mohammadzade G, Mousavi-e-Roknabadi RS, Mohammadzadeh M. Assessment of knowledge, attitudes and practices regarding tuberculosis among final year students in Yazd, central Iran. Journal of Epidemiology and Global Health. 2014;4(2):81–5. doi: 10.1016/j.jegh.2013.09.003 24857175 PMC7366375

[20] Datiko DG, Habte D, Jerene D, Suarez P. Knowledge, attitudes, and practices related to TB among the general population of Ethiopia: Findings from a national cross-sectional survey. PLoS One. 2019;14(10):e0224196. doi: 10.1371/journal.pone.0224196 31658300 PMC6816561

[21] Hoa NP, Chuc NTK, Thorson A. Knowledge, attitudes, and practices about tuberculosis and choice of communication channels in a rural community in Vietnam. Health Policy. 2009;90(1):8–12. doi: 10.1016/j.healthpol.2008.08.006 18835056

[22] Alotaibi B, Yassin Y, Mushi A, Maashi F, Thomas A, Mohamed G, et al. Tuberculosis knowledge, attitude and practice among healthcare workers during the 2016 Hajj. PLoS One. 2019;14(1):e0210913. doi: 10.1371/journal.pone.0210913 30682065 PMC6347151

[23] Mekonnen A, Collins JM, Klinkenberg E, Assefa D, Aseffa A, Ameni G, et al. Tuberculosis knowledge and attitude among non-health science university students needs attention: a cross-sectional study in three Ethiopian universities. BMC Public Health. 2020;20(1):631. doi: 10.1186/s12889-020-08776-5 32375716 PMC7203974

[24] Kamath HS, Vishnu Sharma M, Bhagyalakshmi K, Anupama N. Knowledge, attitude and practice regarding tuberculosis among nursing students in a coastal district of Karnataka. Indian J Tuberc. 2024. doi: 10.1016/j.ijtb.2024.11.004 40975574

[25] Agho KE, Hall J, Ewald B. Determinants of the knowledge of and attitude towards tuberculosis in Nigeria. J Health Popul Nutr. 2014;32(3):520–38. 25395915 PMC4221458

[26] Khan JA, Zahid S, Khan R, Hussain SF, Rizvi N, Rab A, et al. Medical interns knowledge of TB in Pakistan. Trop Doct. 2005;35(3):144–7. doi: 10.1258/0049475054765137 16105336

[27] Aranas LL, Alam K, Gyawali P, Mahumud RA. Drug-Resistant Tuberculosis Stigma Among HealthCare Workers Toward the Development of a Stigma-Reduction Strategy: A Scoping Review. Public Health Rep. 2023;138(4 Suppl):10S-20S. doi: 10.1177/00469580231180754 37310064 PMC10286532

[28] Craig GM, Daftary A, Engel N, O’Driscoll S, Ioannaki A. Tuberculosis stigma as a social determinant of health: a systematic mapping review of research in low incidence countries. Int J Infect Dis. 2017;56:90–100. doi: 10.1016/j.ijid.2016.10.007 27810521

[29] Kılıç A, Zhou X, Moon Z, Hamada Y, Duong T, Layton C, et al. A systematic review exploring the role of tuberculosis stigma on test and treatment uptake for tuberculosis infection. BMC Public Health. 2025;25(1):628. doi: 10.1186/s12889-024-20868-0 39953433 PMC11829483

[30] Xie H, Wang W, Chen X, Huang D, Yu Q, Luo L. An analysis of knowledge, attitudes, practice and influencing factors for tuberculosis prevention and control among Hainan University students. Front Public Health. 2025;13:1478251. doi: 10.3389/fpubh.2025.1478251 39882350 PMC11775896

[31] Ou Y, Luo Z, Mou J, Ming H, Wang X, Yan S, et al. Knowledge and determinants regarding tuberculosis among medical students in Hunan, China: a cross-sectional study. BMC Public Health. 2018;18(1):730. doi: 10.1186/s12889-018-5636-x 29895262 PMC5998553

[32] Launiala A. How much can a KAP survey tell us about people’s knowledge, attitudes and practices? Some observations from medical anthropology research on malaria in pregnancy in Malawi. Anthropol Matters. 2009;11(1).

[33] Rosenstock IM. The Health Belief Model and Preventive Health Behavior. Health Educ Monogr. 1974;2(4):354–86. doi: 10.1177/109019817400200405

[34] Vaidya V, Partha G, Karmakar M. Gender differences in utilization of preventive care services in the United States. J Womens Health. 2012;21(2):140–5. doi: 10.1089/jwh.2011.2876 22081983

[35] Humayun M, Chirenda J, Ye W, Mukeredzi I, Mujuru HA, Yang Z. Effect of Gender on Clinical Presentation of Tuberculosis (TB) and Age-Specific Risk of TB, and TB-Human Immunodeficiency Virus Coinfection. Open Forum Infect Dis. 2022;9(10):ofac512. doi: 10.1093/ofid/ofac512 36324321 PMC9620549

