# Supplementary material for: Tuberculosis-related knowledge, attitudes, and self-reported preventive practices among medical students at the University of Khartoum: A cross-sectional educational survey
Source: PLoS One. 2026 Aug 25;21(8):e0357003. doi: 10.1371/journal.pone.0357003 (PMC13505922; doi:10.1371/journal.pone.0357003)
Supplement: Supplementary file 1 [file pone.0357003.s002.docx]

**Questionnaire on the Tuberculosis-related knowledge, attitudes, and self-reported preventive practices among medical students at the University of Khartoum: a cross-sectional educational survey**

This questionnaire is part of a research study conducted to assess Tuberculosis-related knowledge, attitudes, and self-reported preventive practices among medical students at the University of Khartoum.

The purpose of this study is to better understand students’ awareness and perceptions of tuberculosis and to identify areas that may require further educational interventions and public health attention.

Your participation in this study is entirely voluntary, and you are free to decline participation or withdraw at any time without any consequences. All information collected through this questionnaire will be treated with strict confidentiality and used solely for academic and research purposes. No personally identifiable information will be collected, and responses will remain completely anonymous.

###### **-Do you agree to participate in this study?**

Yes 🞏	No 🞏

**Section 1: Socioemographic characteristics**

**1. Age:**

≤22 🞏

>22 🞏

**2. Sex:**

Male 🞏

Female 🞏

###### **3. Year of Study:**

Third year (Batch 98) 🞏

Fourth year (Batch 97) 🞏

Fifth year (Batch 96) 🞏

Sixth year (Batch 95) 🞏

###### **4. Have you ever heard about tuberculosis?**

Yes 🞏

No 🞏

###### **5. Source of Information (you can choose multiple responses):**

Social media 🞏

Formal education 🞏

Internet 🞏

TV 🞏

Healthcare providers 🞏

Others 🞏

###### **Section 2: Knowledge about Tuberculosis**

**1. What causes TB?**

Bacteria 🞏

Virus 🞏

Fungus 🞏

Don’t know 🞏

###### **2. Which of the following are common symptoms of pulmonary TB? (you can choose multiple responses)**

Fever 🞏

Rash 🞏

Cough lasting more than 2 weeks 🞏

Weight loss and loss of appetite 🞏

Blood in sputum 🞏

Chest pain 🞏

Night sweats 🞏

Abdominal pain 🞏

Don’t know 🞏

###### **3. How can a person get TB?**

Through air droplets during coughing or sneezing 🞏

Through handshakes 🞏

Sexually transmitted 🞏

Orally through unclean food and water 🞏

Don’t know 🞏

###### **4. In which region of the lung do pulmonary tuberculosis lesions typically occur?**

Lower lobes 🞏

Middle lobes 🞏

Upper lobes 🞏

Don’t know 🞏

###### **5. Patients with latent tuberculosis can spread the disease:**

True 🞏

False 🞏

Don’t know 🞏

###### **6. What is the most appropriate diagnostic method for pulmonary TB?**

Blood test 🞏

Sputum test 🞏

Chest X-ray 🞏

Urine/stool test 🞏

Don’t know 🞏

###### **7. What does a positive tuberculin skin test for TB indicate?**

It indicates a latent TB infection only. 🞏

It indicates active TB disease only. 🞏

It indicates either a latent TB infection or active TB disease. 🞏

Don’t know 🞏

###### **8. BCG vaccine is administered to newborns as part of their vaccination schedule:**

True 🞏

False 🞏

Don’t know 🞏

###### **9. What is the mainstay of treatment for most patients with pulmonary tuberculosis?**

Ciprofloxacin 🞏

Isoniazid, rifampicin, pyrazinamide, ethambutol 🞏

Ceftriaxone, cefixime 🞏

Don’t know 🞏

###### **10. How long does it take to treat TB?**

1–2 weeks 🞏

1–2 months 🞏

6–8 months 🞏

1–2 years 🞏

Don’t know 🞏

**Section 3. Attitudes toward tuberculosis**

| **Attitude questions** | **Strongly Agree** | **Agree** | **Neutral** | **Disagree** | **Strongly Disagree** |
| --- | --- | --- | --- | --- | --- |
| 1. **I feel uncomfortable when anybody nearby sneezes or coughs without covering their mouth and nose** |  |  |  |  |  |
| 2. **A person should be examined for TB if there is a TB patient among their family or friends.** |  |  |  |  |  |
| 3. **I can’t imagine myself working with tuberculosis (TB) in the future.** |  |  |  |  |  |
| 4. **I think TB can be cured if it is detected and treated early.** |  |  |  |  |  |
| 5. **Quarantine of TB patients may reduce the risk of the spread of TB.** |  |  |  |  |  |
| 6. **I believe that educating people about TB prevention methods is crucial for reducing the risk of TB and improving overall public health.** |  |  |  |  |  |
| 7. **TB-related stigma affects individuals' willingness to seek medical care and treatment.** |  |  |  |  |  |

**Section 4. Preventive practices toward tuberculosis**

| **Practice questions** | **Always** | **Often** | **Sometimes** | **Rarely** | **Never** |
| --- | --- | --- | --- | --- | --- |
| 1. **I cover my mouth when I cough or sneeze.** |  |  |  |  |  |
| 2. **I wear a face mask or other appropriate respiratory protection when caring for or interacting with patient suspected or confirmed to have TB** |  |  |  |  |  |
| 3. **I perform hand hygiene and wear personal protective equipment before contact with pulmonary TB patients.** |  |  |  |  |  |
| 4. **I open windows when possible in TB patients' rooms to increase natural ventilation.** |  |  |  |  |  |
| 5. **I read materials designed to raise awareness about TB and respiratory infections.** |  |  |  |  |  |
