# Supplementary material for: Tuberculosis-related knowledge, attitudes, and self-reported preventive practices among medical students at the University of Khartoum: A cross-sectional educational survey
Source: PLoS One. 2026 Aug 25;21(8):e0357003. doi: 10.1371/journal.pone.0357003 (PMC13505922; doi:10.1371/journal.pone.0357003)
Supplement: Supplementary file 2 [file pone.0357003.s003.docx]

# STROBE Statement Checklist for PLOS ONE Submission

**Manuscript:** Tuberculosis-related knowledge, attitudes, and self-reported preventive practices among medical students at the University of Khartoum: a cross-sectional educational survey

| Item | Recommendation | Reported on Page(s) | Status |
| --- | --- | --- | --- |
| 1 | Title and abstract | 1-2 | Yes |
| 2 | Background/rationale | 3 | Yes |
| 3 | Objectives | 3 | Yes |
| 4 | Study design | 4 | Yes |
| 5 | Setting | 4 | Yes |
| 6 | Participants | 4-5 | Yes |
| 7 | Variables | 5-6 | Yes |
| 8 | Data sources/measurement | 5-6 | Yes |
| 9 | Bias | 6 | Yes |
| 10 | Study size | 4 | Yes |
| 11 | Quantitative variables | 5-6 | Yes |
| 12 | Statistical methods | 6 | Yes |
| 13 | Participants/results | 7 | Yes |
| 14 | Descriptive data | 7 | Yes |
| 15 | Outcome data | 7-13 | Yes |
| 16 | Main results | 11-13 | Yes |
| 17 | Other analyses | 11-13 | Yes |
| 18 | Key results | 13-16 | Yes |
| 19 | Limitations | 15-16 | Yes |
| 20 | Interpretation | 13-16 | Yes |
| 21 | Generalisability | 15-16 | Yes |
| 22 | Funding | In submission system | Yes |
